# The Antioxidant Potential of Commercial Manuka Honey from New Zealand—Biochemical and Cellular Studies

**DOI:** 10.3390/cimb46070380

**Published:** 2024-06-25

**Authors:** Julia Kaźmierczak-Barańska, Bolesław T. Karwowski

**Affiliations:** DNA Damage Laboratory of Food Science Department, Faculty of Pharmacy, Medical University of Lodz, Ul. Muszynskiego 1, 90-151 Lodz, Poland; julia.kazmierczak-baranska@umed.lodz.pl

**Keywords:** manuka honey, polyphenols, DPPH, methylglyoxal, antioxidant, UV radiation, cell cultured

## Abstract

Manuka honey (MH) is considered a superfood mainly because of its various health-promoting properties, including its anti-cancer, anti-inflammatory, and clinically proven antibacterial properties. A unique feature of Manuka honey is the high content of methylglyoxal, which has antibacterial potential. Additionally, it contains bioactive and antioxidant substances such as polyphenols that contribute to its protective effects against oxidative stress. In this study, commercially available Manuka honey was tested for its total polyphenol content and DPPH radical scavenging ability. It was then tested in vitro on human fibroblast cells exposed to UV radiation to assess its potential to protect cells against oxidative stress. The results showed that the honey itself significantly interfered with cell metabolism, and its presence only slightly alleviated the effects of UV exposure. This study also suggested that the MGO content has a minor impact on reducing oxidative stress in UV-irradiated cells and efficiency in scavenging the DPPH radical.

## 1. Introduction

Manuka honey is made from the flower nectar of the *Leptospermum scoparium* tree, native to New Zealand and south-east Australia, and is harvested primarily in New Zealand. The main flavonoids in Manuka honey are chrysin, pinobanksin, and pinocembrin. Chrysin occurs not only in honey and propolis but also in natural plant sources and some edible mushrooms, such as the oyster mushroom (*Pleurotus ostreatus*) [1,2,3]. Chrysin has antioxidant, anti-inflammatory, antihepatotoxic, and anticancer effects [1,4], which have been linked to its free radical scavenging function and increased superoxide dismutases (SOD), catalase (CAT), glutathione peroxidase (GPx3), and glutathione s-transferase (GST) gene expression under conditions of oxidative stress [5]. Chrysin may have a neuroprotective effect, which is related to its ability to reduce oxidative stress by influencing the biosynthesis of sphingolipids [6]. Pinobanksin is one of the common flavonoids present in propolis [7], which was shown to be anti-angiogenic, leading to a significant reduction in cell viability, inhibition of cell migration, and tube formation in endothelial cells. Further studies showed that this is the result of apoptosis induction. These properties of pinobanksin make it a potentially useful agent in preventing tumor growth [8]. Propolis is also a rich source of pinocembrin. However, pinocembrin has been isolated from many plant species [9], and studies have shown that pinocembrin has an effect that limits the accumulation of amyloid β in brain tissue, reduces the permeability of the blood–brain barrier, delays neuronal apoptosis, and, of course, has an antioxidant effect [10,11,12,13]. The human body is exposed to different types of electromagnetic radiation from natural and artificial sources. Radiation is widely used in industry and medicine and contributes to a significant increase in environmental pollution. Similarly, excessive exposure to UV radiation triggers the generation of oxidative stress and cell and DNA damage, with particularly harmful effects observed for the skin and eyes. Despite this, UV rays fortunately do not have enough energy to penetrate and damage organs deep in the human body. Since radiation-induced cell damage is primarily attributed to the damaging effects of free radicals, molecules with radical scavenging properties are particularly promising as radioprotectants [14,15]. It is generally recognized that consuming natural substances containing antioxidants can reduce the risk of damage by scavenging free radicals from biological systems. An epidemiological study conducted among the population of Hiroshima and Nagasaki who survived the nuclear explosions showed that even after exposure to a high dose of radiation (mean 0.1 Sv), a diet rich in vegetables and fruits, being rich sources of anticancer compounds such as antioxidants, significantly reduced (up to 50%) the risk of cancer [16]. Various polyphenolic compounds with antioxidant properties known to protect cell structures and genetic material against irradiation (IR) have been identified in honey. In recent years, there has been an increase in interest in Manuka honey, whose anti-cancer and antioxidant potential and pro-health, anti-inflammatory, antibacterial, antiviral, wound-healing, and immunostimulating properties are described in the literature [17,18,19,20,21]. As a result, because of its favorable nutritional and phytochemical profile, compared with, e.g., buckwheat or multifloral varieties, Manuka honey is becoming increasingly popular in pharmacies and “health food” stores. However, with growing interest, this expensive dietary supplement has become subject to widespread counterfeiting.

Methylglyoxal (MGO), also known as acetylformaldehyde, 2-ketopropionaldehyde, pyruvaldehyde, is a unique compound contained in Manuka honey that determines its strong antibacterial properties. MGO is believed to be an indicator of stress accompanying unfavorable plant growth conditions. MGO is cytotoxic to plant cells at high cellular concentrations, but at low concentrations, it acts as an important signaling molecule enabling some plants to grow and develop under environmental stress [22]. Manuka honey has a unique antibacterial effect that distinguishes it from other types of honey. While most honey produces hydrogen peroxide through glucose oxidase, Manuka honey has low glucose oxidase activity and instead contains methylglyoxal, which is responsible for its “non-peroxide activity” (NPA) [23,24,25,26]. The presence of methylglyoxal makes Manuka honey an effective treatment for antibiotic-resistant bacteria such as *Helicobacter pylori* [27]. The antibacterial mechanism of action of methylglyoxal is related to its ability to induce the glycation process and cross-linking of lysine and arginine residues, which leads to the inactivation of bacterial proteins [28]. Methylglyoxal is also present in other types of honey and food products but in significantly lower amounts [29,30]. Terio et al. report MGO levels ranging from 6.8 to 17 mg/kg in thyme honey and from 6.3 to 18.4 mg/kg in multifloral honey. Nevertheless, it is unclear whether more means better in every case. The present study aimed to evaluate the potential protective role of Manuka honey against UV-induced fibroblast damage.

## 2. Materials and Methods

### 2.1. Honey Samples

Four samples of commercially available Manuka honey (New Zealand) were obtained from Propharma (Poland). According to the manufacturer’s labeling, the minimum MGO levels in the honey samples were 30 mg/kg (MGO30), 100 mg/kg (MGO100), 250 mg/kg (MGO250), and 550 mg/kg (MGO550). All honey samples were kept at room temperature (23 ± 2 °C) throughout the analysis. By analyzing the amount and morphology of plant pollen in the samples, the source, origin, and species of honey were determined. The honey variety was determined in accordance with the methodology specified in the Regulation of the Minister of Agriculture and Rural Development (Poland), of 14 January 2009, (according to the Journal of Laws of 2009, No. 17, item 94, as amended, Journal of Laws of 2015, item 1173), by examining the percentage of predominant pollen (%p), which was calculated according to the formula:

%p = (Ndp × 100)/Np

where Ndp is the number of pollen grains of the dominant plant and Np is the number of pollen grains of nectar-producing plants.

### 2.2. Determination of Total Polyphenols

The total levels of phenolic compounds (TP) were estimated using Folin–Ciocalteu’s phenol reagent (Alpha Chemical, Navi Mumbai, India) according to a modified Folin–Ciocalteu method [31]. Gallic acid (Sigma Aldrich, St. Louis, MO, USA) was used to generate the standard curve. Samples were analyzed in quadruplets. Briefly, 1 mL of Manuka honey solution (100 mg/mL, dilution in distilled water) from each sample was mixed with 5 mL of Folin–Ciocalteu’s phenol reagent 10%. After 5 min, 4 mL of 7.5% sodium bicarbonate was added to the solution. The mixture was maintained at room temperature in the dark for 30 min, after which the absorbance was recorded at 765 nm using a spectrophotometer. The total phenolic content was calculated using the calibration curve generated from standard solutions of gallic acid ranging from 10 to 70 μg/mL. The results were expressed as mean ± standard deviation and presented in milligrams of gallic acid equivalents (GAEs).

### 2.3. Free Radical Scavenging Activity

Free radical scavenging activity was determined by the 2,2-diphenyl-1-picrylhydrazyl (DPPH) assay (Sigma Aldrich). Briefly, 100 µL of Manuka honey (100 mg/mL) from each sample was mixed with 0.5 mL of DPPH (0.052 mg/mL in methanol). The reaction mixtures were well mixed and incubated in the dark at room temperature for 30 min, after which the absorbance (A) was measured at λ 517 nm by a VIS 6000 (Kruss, Hamburg, Germany) spectrophotometer. The RSA was calculated as a percentage of DPPH discoloration using the equation:

% RSA = [(A_DPPH_ − A_S_)/A_DPPH_] × 100

where A_S_ is the absorbance of the sample solution and A_DPPH_ is the absorbance of the DPPH solution (in the absence of Manuka honey). All measurements were performed in triplicate.

### 2.4. Cell Culture and MTT Assay

Fibroblasts are the basic element of connective tissue and form the skin or mucous. Skin fibroblast cells were selected for the present study because the skin, the largest external organ, is exposed to various types of radiation. The human fibroblast BJ (CRL-2522) cell line was obtained from the American Type Culture Collection (Rockville, MD, USA). The cells were cultured in basic Minimum Essential Medium (MEM) supplemented with 10% fetal bovine serum and 100 units per mL penicillin and streptomycin antibiotic mixture at 37 °C in a humidified 5% CO_2_ atmosphere. The cells were cultured (passage 2 for post-cryopreserved cells) and passaged for up to eight passages under standard conditions as described above.

The cells were divided into six groups (n = 6/group). Group C included cells cultivated under standard conditions without UVB exposure, which acted as the control. Group IR included cells cultivated under standard conditions but exposed to UVB radiation. Group H-IR included cells pre-treated with honey for 24 h and then exposed to UVB irradiation. Group IR-H included cells exposed to UVB irradiation and then post-treated with honey for 24 h. Similarly, two other groups of cells were treated with honey in the same way but not UVB-irradiated, i.e., the honey pre-treated group (group pre-H) and the honey post-treated (group post-H) group. All “H” cultures were incubated for 24 h with 1 g/mL of Manuka honey (*w*/*v* in PBS), after which the medium was removed and replaced with fresh medium before adding the MTT reagent.

The cells were irradiated with a wavelength of 302 nm and an irradiance of 150 W/cm^2^ for 30 s. A single well in the plate had an area of 0.32 cm^2^, and the total power of the UV lamps used was 48 W. The radiation dose density was 4500 J/cm^2^. Cellular metabolic activity was determined using the colorimetric 3-(4,5-dimethyl-2-thiazolyl)-2,5-diphenyl-2-H-tetrazolium bromide (MTT) assay. This assay has a wide range of applications as a test of the metabolic activity of cells because the MTT reagent, after passing through the cell membrane and the mitochondrial membrane of living cells, is reduced to formazan by metabolically active cells [32]. Briefly, 25 mL MTT solution (5 mg/mL) in phosphate-buffered saline (PBS) buffer (155 mM NaCl, 4.2 mM KH_2_PO_4_/Na_2_HPO_4_, pH 7.2) was added to each well and incubated for four hours at 37 °C; the cells were then continuously observed under a phase-contrast microscope. Finally, 95 mL lysis buffer (20% SDS, 50% aqueous dimethylformamide, pH 4.5) was added to each well and incubated at 37 °C for an additional 24 h. The absorbance of a given sample was measured at 570 nm, with the reference wavelength 630 nm (Microplate Reader 450, Bio-Rad, Hercules, CA, USA). The relative metabolic activity of cells (RMA) was calculated from the equation:

RMA = [(AS − AM)/(AC − AM)] × 100%

where AS is the absorbance of a given sample of cells treated with Manuka honey, AM is the absorbance of a cell medium, and AC is the absorbance of control (Manuka untreated: C or IR, respectively, for unexposed or exposed UV variants) cells [33]. Data points represent mean values from three independent experiments (each with six replicates). For the purpose of comparison, we used the preliminary calculations to make the IR group our reference point (i.e., 1) and compared other results to it.

### 2.5. Statistical Analysis

The statistical analysis was performed using Statistica for Windows 10.0. The results were presented as means and standard deviations. One-way analysis of variance (ANOVA) using Tukey’s post-test was performed, and different letters in the same row or column were used in tables to indicate statistical significance (at *p* < 0.05). In the fibroblast studies, statistical analysis was performed using two-way analysis of variance (ANOVA) to assess significant differences between different groups. The results were considered significant when *p* < 0.05.

## 3. Results

### 3.1. Estimation of Predominant Pollen

**Figure 1:**
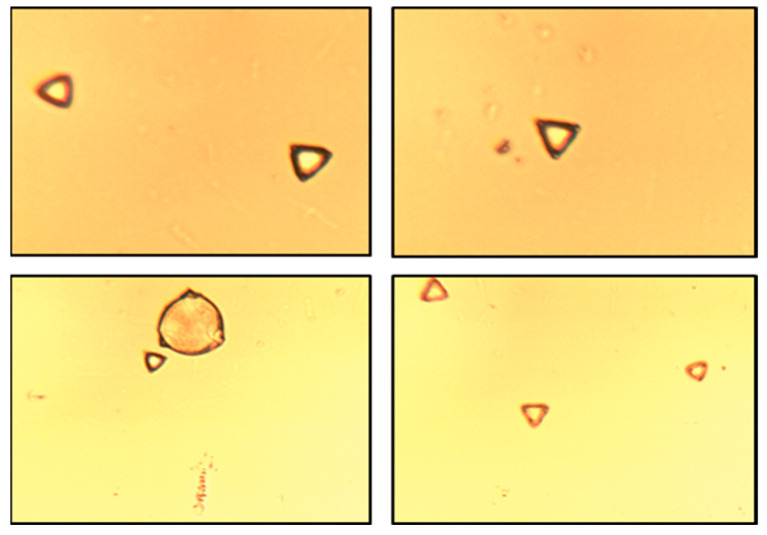
Microscopic photographs (magnification: 40×) of pollen grains.

The amount of predominant pollen was estimated (from total pollen) at 80%, 89%, 50%, and 91%, respectively, for 30 mg/kg, 100 mg/kg, 250 mg/kg, and 550 mg/kg Manuka honey samples. The morphology of Manuka pollen (Figure 1) was determined based on the literature data [34,35]. These results confirm the variety of honey and that the honey used for further research was not artificial or counterfeit.

### 3.2. Total Phenol Contents and Antioxidant Activity of Honey

The total level of phenolic compounds (TP) was around 53.5 mg GAE/100 g of honey (Table 1). The highest TP content was found in MGO550 Manuka honey (56.75 mg GAE/100 g) and the lowest in MGO30 honey (52.53 mgGAE/100 g). Antioxidant activity was defined as the ability to scavenge DPPH radicals by antioxidants contained in Manuka honey, measured by the DPPH test. These results indicate that the antioxidant activity ranged from 38.67% for MGO30 honey to 50.36% for MGO550 honey. In addition, the Manuka honey variety with the highest MGO content (MGO550) also demonstrated the highest total polyphenol content and the highest DPPH free radical scavenging activity.

### 3.3. Radioprotective Effect of Manuka Honey

**Figure 2:**
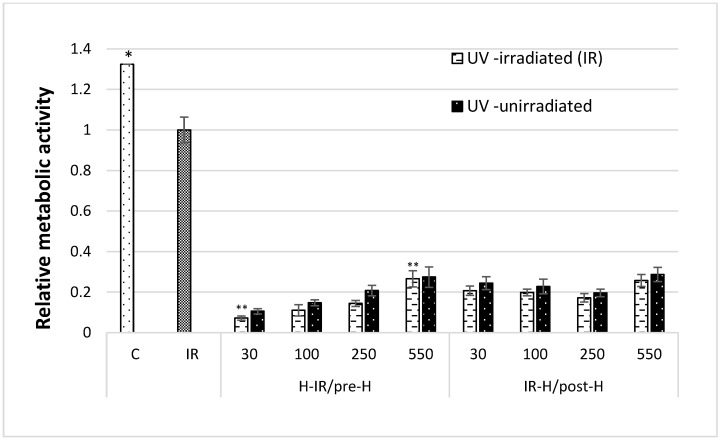
The metabolic rate of fibroblasts in the presence of different content of MGO in Manuka honey. C: cells cultivated in standard conditions without UV exposure, control. IR: cells cultivated in standard conditions were exposed to UV radiation. H-IR: cells were pre-treated with Manuka honey for 24 h and then exposed to UV irradiation. IR-H: cells exposed to UV irradiation and then post-treated with Manuka honey for 24 h. Two groups of cells were treated with honey in the same way but not irradiated with UV, i.e., the pre-treated (pre-H) group and the post-treated (post-H) group. All data represent the result of three different experiments (mean ± SEM). * Tested groups compared with group C (*p* < 0.05). ** H-IR 30 compared with H-IR 550 (*p* < 0.05).

The analysis of control groups, i.e., cells not treated with honey (groups C and IR), found a 30% decrease in the metabolic activity of the cells as a result of irradiation. Similarly, among the cells treated with the tested honey samples, those treated with UV demonstrated lower metabolic activity than the unirradiated cells (Figure 2). The protective effect of honey was more clearly visible in the group incubated after UV irradiation (IR-H compared with post-H), where the metabolic rate after UV radiation decreased by 15% for 30 mg/kg MGO honey, 13% for 100 mg/kg MGO, 13% for 250 mg/kg MGO, and 10% for 550 mg/kg MGO.

In the group of cells incubated with honey before irradiation (H-IR compared with pre-H), UV treatment reduced the metabolic rate by approximately 30% (33% for 30 mg/kg MGO, 25% for 100 mg/kg MGO, and 31% for 250 mg/kg MGO). An exception in this group was MGO550 honey, which had the strongest protective effect: the cell metabolism rate decreased by only 5% after UV exposure. There was a significantly lower decrease in metabolic rate (** *p* < 0.05) in the group preincubated with MGO550 honey and exposed to UV radiation (H-IR 550) compared with the group exposed to UV radiation and preincubated with MGO30 honey (H-IR 30). An interesting fact is that Manuka honey with an MGO content of at least 550 mg/kg in each experimental variant (H-IR, IR-H) showed the best effectiveness in alleviating the negative effects of UV radiation among the tested honey samples. MGO550 honey also showed the highest efficiency in scavenging the DPPH radical (50.36%) and the highest total polyphenol content (TP: 56.75 mg/100 g). It is worth noting that all honey varieties caused strong and significant inhibition of cell metabolism (* *p* < 0.05). The honey itself significantly interfered with cell metabolism, and its presence only slightly alleviated the effects of UV exposure. The MGO content seems to be of rather little importance.

## 4. Discussion

The literature suggests that polyphenols, which play a crucial role in reducing oxidative stress, are also a factor in the antioxidant potential of honey. In other words, the content of polyphenolic compounds may, to some extent, reflect the total antioxidant activity of honey [36]. In a study conducted by Goslinski et al. [19], Manuka honey had a high total polyphenol content (over 240 mg GAE/100 g), but lower values were found in Wilczyńska [37] (149.5 mg GAE/100 g) and Mokaya et al. (94 mg GAE/100 g) [38]. Alzahrani et al. determined an even lower total polyphenol content in Manuka honey, i.e., 89.9 mgGAE/100 g [39]. We found a relatively low TP content in Manuka honey (56 mg GAE/100 g for MGO 550). Similarly, the value of TP reported by Hulea et al. was approximately 53 mg GAE/100 g [40]. A similar observation applies to ROS scavenging activity. Our study showed that the DPPH radical scavenging activity was 50.36% of RSA for MGO550 honey, which had the highest TP content. Wilczyńska reported a value of 74.07% RSA for MGO550 honey [39], while Hunter et al. [41] determined the percentage of DPPH radical inhibition at 69% RSA, as did Hulea et al. (68%RSA) [42]. Comparing and discussing the results is hampered by the significant differences in the results reported by various research groups. These differences may be due to the use of different samples, as environmental factors, age, and storage methods can affect the quality of honey batches. The presented results suggest that the concentration of MGO has only a negligible effect on the content of polyphenols (TP) and %RSA in Manuka honey.

MGO is also a natural metabolic product of human cells, formed mainly as a by-product of anaerobic glycolysis, which is responsible for the glycation of amino acids (arginine) or nucleotides (dG/G). Excessive cellular concentrations of MGO lead to protein inactivation and dysfunction, thus potentially leading to problems with apoptosis and replication [42]. High concentrations of exogenous MGO or the use of Glo1 (methylglyoxal metabolizing enzyme) inhibitors can induce cell death or cell cycle arrest [43,44,45]. Kani et al. demonstrated that both the mitogen-activated protein kinase (ASK1-JNK/p38 MAPK) and checkpoint kinase (ATM-Chk1/Chk2) pathways are involved in MGO-induced cell cycle arrest and/or the apoptosis of cultured cells via oxidative stress signaling [45]. Furthermore, Sjersen et al. conducted research indicating that treating human skin fibroblasts with 400 µM of MGO leads to the acceleration of cellular aging. MGO treatment has been shown to lead to irreversible growth arrest, increased senescence-associated β-galactosidase (SABG) activity, and increased levels of Nξ-(carboxymethyl)-lysine (CML), which are recognized molecular markers of aging [46]. This may explain the strong decrease in the metabolic activity of the fibroblasts incubated with Manuka honey observed in the present study, which showed a decrease of 80–90% in the pre-H and post-H groups compared with the controls (group C). Its antioxidant properties allow for the scavenging of free radicals [47] that are normally involved in cell signaling to a small degree [48,49], for example, reactive oxygen species (ROS) appear to be required for intracellular signaling involved in central nervous system (CNS) plasticity [50]. In addition, as part of the Epidermal Growth Factor (EGF) signaling pathway, ROS are involved in growth factor-induced phosphorylation of the EGFR receptor [51]. It is therefore not surprising that the use of a highly potent scavenger also causes the inhibition of metabolism in non-irradiated cells. Our findings suggest that Manuka honey inhibits the metabolism of fibroblasts and prevents them from dividing. This information is noteworthy because most studies focus on the anti-cancer properties of Manuka honey, while less information is available on its impact on normal cell lines. Among the different variants of Manuka honey tested, the one with the highest TP content and DPPH scavenging activity (MGO content of at least 550 mg/kg) showed the strongest effectiveness in alleviating the harmful effects of UV radiation. However, our findings suggest that the concentration of MGO is not a significant factor in determining the antioxidant activity or the potential to alleviate the effects of oxidative stress.

Hence, MGO seems to be a somewhat ambiguous factor that offers strong antibacterial potential beneficial against infectious diseases and also cytotoxic potential. The presented results are interesting from the point of view of wound healing. Manuka honey is available in the form of an ointment that supports the healing of skin damage from various etiologies [52]. As mentioned above, free radicals are involved in the activation of fibroblasts, which determines the repair process; however, if inflammation (including the production of reactive oxygen species) persists, then overstimulation can lead to fibrosis and excessive production of collagen fibers [53]. Nevertheless, Majtan [54] points to the role of MGO in the impaired healing of diabetic wounds. Although clinical studies demonstrate the benefits of using Manuka honey for treating acute wounds such as burns, it is important to note that high concentrations of MGO may not be beneficial for diabetic ulcers and may even have a harmful effect.

Based on previous reviews and meta-analyses, honey is considered to be a health-promoting product because of its high content of various bioactive substances. It has the potential to improve the quality of life (QoL) of radiological patients and reduce the side effects of mucositis [55,56,57,58]. According to the research, honey is characterized by high bioavailability and bioactivity and can strengthen defense mechanisms against oxidative stress—an increase in the plasma phenolic content and plasma antioxidant capacity was observed in tested volunteers after consuming honey [59,60]. However, it remains unclear whether patients or consumers should choose Manuka honey instead of local honey varieties. A systematic review of randomized trials by Münstedt et al. on the potential of honey to alleviate the side effects of radiotherapy found positive results for conventional honey but none for Manuka honey, which was often poorly tolerated by patients [61]. The probable cause may be high concentrations of MGO. The results presented by Zhang et al. indicate that MGO may cause a wide range of systemic symptoms of irritable bowel syndrome (IBS), such as diarrhea, abdominal pain, headache, depression, cognitive impairment, arrhythmia, and skin problems [62]. Exogenous sources of MGO, although absorbed partially, do not accumulate entirely in tissues as free MGO. Instead, MGO rapidly reacts to produce in situ adducts, also known as advanced glycated end products (AGEs). Depending on the specific protein’s nature and half-life, these AGEs may remain confined to the site of administration or translocate in various ways to other parts of the body. MGO-derived AGEs can be harmful to macrovascular and microvascular functions, causing nephropathy and neuropathy [63].

Samples that are used for laboratory testing and research are generally collected from beekeepers and stored according to the highest standards to ensure that the honey analyzed is of the highest quality and has not been industrially processed. However, when it comes to commercial distribution, the same level of standards may not always be upheld. The demand for New Zealand Manuka honey and its pro-health benefits has led to a substantial rise in its price, making it vulnerable to various types of fraud [64]. It is important for consumers to be informed and educated about the benefits of choosing local and seasonal products, and whenever possible, to obtain them directly from beekeepers. Imported products from distant parts of the world are often more susceptible to harmful factors such as light and temperature during transportation and storage, which can negatively affect the quality of this expensive product.

## 5. Conclusions

In summary, the results presented in our study indicate that the concentration of MGO has a negligible impact on the content of polyphenols (TP) and %RSA in Manuka honey. MGO is not an important factor in determining the antioxidant activity or the potential to alleviate the effects of oxidative stress. Our findings also suggest that Manuka honey inhibits the metabolism of fibroblasts and prevents cell division. This information is significant because limited data are available on the effects of Manuka honey on normal cell lines. However, our study suggests that further research is necessary to establish the safe medical uses of Manuka honey.

## Figures and Tables

**Table 1:** Total phenol contents and antioxidant activity of honey. The values of TP are expressed as mg/100 g for honey. The results are expressed as mean ± SD.

Sample *	Total Polyphenol [mgGAE/100 g]	%RSA
30+	52.53 ± 0.26 ^a^	38.67 ± 0.52 ^a^
100+	53.6 ± 0.30 ^b^	43.71 ± 0.57 ^b^
250+	53.48 ± 0.12 ^b^	46.64 ± 0.15 ^c^
550+	56.75 ± 0.108 ^c^	50.36 ± 0.14 ^d^

* Each variant of Manuka honey contained different amounts of methylglyoxal. Data represent the mean ± standard deviation (*n* = 4). Statistical analysis was performed by one-way ANOVA using Tukey’s post hoc test: different letters in the same column indicate statistical significance (*p* < 0.05).

## Data Availability

The datasets presented in this article are not readily available due to technical limitations. Requests to access the datasets should be directed to julia.kazmierczak-baranska@umed.lodz.pl.
